# Evaluation and validation of suitable reference genes for quantitative real-time PCR analysis in lotus (*Nelumbo nucifera* Gaertn.)

**DOI:** 10.1038/s41598-024-61806-9

**Published:** 2024-05-13

**Authors:** Bin Wang, Fenglin Zhu, Xingwen Zheng, Liangbo Yang, Ying Diao, Zhongli Hu

**Affiliations:** 1 College of Life Science and Technology, Wuhan Polytechnic University, Wuhan, 430023 People’s Republic of China; 2 Anhui University of Science and Technology, Medical College, Huainan, 232001 People’s Republic of China; 3 State Key Laboratory of Hybrid Rice, Lotus Engineering Research Center of Hubei Province, College of Life Science, Wuhan University, Wuhan, 430072 People’s Republic of China; 4 Guangchang County White Lotus Industrial Development Center, Guangchang, 344900 P.R. China

**Keywords:** Lotus, Reference gene, qRT-PCR, Gene expression, geNorm, NormFinder, Biological techniques, Molecular biology, Plant sciences

## Abstract

The qRT-PCR technique has been regarded as an important tool for assessing gene expression diversity. Selection of appropriate reference genes is essential for validating deviation and obtaining reliable and accurate results. Lotus (*Nelumbo nucifera* Gaertn) is a common aquatic plant with important aesthetic, commercial, and cultural values. Twelve candidate genes, which are typically used as reference genes for qRT-PCR in other plants, were selected for this study. These candidate reference genes were cloned with, specific primers designed based on published sequences. In particular, the expression level of each gene was examined in different tissues and growth stages of Lotus. Notably, the expression stability of these candidate genes was assessed using the software programs geNorm and NormFinder. As a result, the most efficient reference genes for rootstock expansion were TBP and UBQ. In addition, TBP and EF-1α were the most efficient reference genes in various floral tissues, while ACT and GAPDH were the most stable genes at all developmental stages of the seed. CYP and GAPDH were the best reference genes at different stages of leaf development, but TUA was the least stable. Meanwhile, the gene expression profile of NnEXPA was analyzed to confirm the validity of the findings. It was concluded that, TBP and GAPDH were identified as the best reference genes. The results of this study may help researchers to select appropriate reference genes and thus obtain credible results for further quantitative RT-qPCR gene expression analyses in Lotus.

## Introduction

One of the most common molecular techniques used to confirm the function of candidate genes is gene expression pattern analysis^1^. Therefore, qRT-PCR (quantitative real-time reverse transcription polymerase chain reaction) is increasingly favoured in gene expression analysis as a highly sensitive and accurate expression profiling technique^2^. Introduced in 1992, qRT-PCR can detect such a small number of mRNA copies, display Ct values, and enable the sample to be expressed^3^. Undoubtedly, qRT-PCR has the advantages of real-time detection of the reaction process, fast analysis speed, and high sensitivity to accurately measure the detected substances in the sample. However, the accuracy of qRT-PCR is significantly affected by RNA integrity, cDNA quality and qRT-PCR amplification efficiency^4^. Among the strategies for normalising qRT-PCR data to accurately quantify gene expression, the method of normalisation with one or more reference genes is widely used^5^. This method, like traditional mRNA quantification methods, requires normalisation, i.e., a reference gene^6^, to ensure the reliability and accuracy of the quantitative result. For qRT-PCR, it is necessary to select the applicable reference gene to avoid some common problems. Gene expression patterns using a reference gene as a standard will show small differences in gene expression in different tissues or cells of an organism and in different physiological states.

Ideal reference genes should be stable in all organ and physiological states and be able to be used in a variety of samples. Therefore, we selected some HKGs (housekeeping genes) as reference genes for qRT-PCR. Some are essential components of the organelle skeleton, such as *ACT*, *18S* and *TUA*; some are involved in the basic biochemical metabolic processes of the organism, such as *EF-1α*, *UBC and GAPDH*^7^. However, none of the reference genes are always stable with changing experimental conditions^8^. Many studies have reported that the applicability of these reference genes used for normalisation in real-time PCR has not been verified in any way, and that the reference genes are not necessarily equally applicable to other genes^9^. Subsequently, they found that this variation may occur in different species, tissues, experiments or specific stress treatments. qRT-PCR has demonstrated that *UBC* exhibits different expression patterns in different tissues of Lotus^10^. Therefore, accurate reference genes are necessary to distinguish the expression of closely related genes and to quantify the transcript levels of very weakly expressed genes, even if two or more reference genes need to be used. Selection of appropriate reference genes was made through the geNorm and NormFinder algorithm software, which were recently developed to determine the best reference genes to use under specific experimental conditions^11,12^.

Lotus (*Nelumbo nucifera* Gaertn) is an import aquatic vegetable, that has been cultivated and domesticated in almost all provinces of China for more than 2000 years^13^. The rhizomes and seeds of Louts have the highest nutrient content among the twelve aquatic vegetables, including starch, protein, several vitamins and secondary metabolites^14^. Therefore, it is used not only as a vegetable but also as a medicinal herb, tea, and dessert. Lotus is beneficial to the food economy, hence more and more research has been done on it recently^15,16^, including transcriptome, genome, polymorphic markers and gene identification^17,18^. However, there is no scientific analysis on the selection of normalised reference genes in different developmental stages and stress treatments in Lotus.

In the study, 12 reference genes (*18S, ACT, CYP, UBQ, UBC, TUA, GAPDH, EF-1α, MDH, PLA, TBP, Eif-5a*) were selected and tested in different tissues of Lotus to obtain one or more candidate reference genes for qRT-PCR. Notably, the achieved results may provide valuable information for gene expression studies in lotus.

## Materials and methods

### Plant material

The *Nelumbo nucifera* cv. lotus cultivar Tai-Kong Lian No. 36 was grown in Wuhan University’s greenhouse in Hubei Province, China. Sprouted seeds were placed in the pots after 3 days of germination under the growth conditions of sixteen hours light and eight hours dark, and room temperature at 25 °C. The tissues that were examined include leaves (initial leaf, young leaf, mature leaf), rhizome (initial rhizome, swelling rhizome, stolon), seeds (four developing stages: cell division; of cell vacuolization; physiological accumulation; maturation), flowers (bud, perianth, seedpod, pericarp, anther, thrum, carpel), root, and stalk. All samples were collected from three replicate plants and frozen in liquid nitrogen immediately, then stored at − 80 °C until RNA extraction.

### RNA isolation and cDNA synthesis

RNA was extracted from lotus tissue employing the TIANGEN RNAprep Plant Kit (China) adhering to the manufacturer’s instructions. The use of PVP K30 (Polyvinyl Pyrrolidone) during grinding was essential to eliminate polysaccharides and polyphenols, given the unique of the lotus. To ensure gDNA contamination was minimized, all RNA samples underwent treatment with RNase-free DNase I. The integrity of the RNA was assessed through 1.2% agarose gel electrophoresis. Subsequently, cDNA (complementary DNA) was synthesized using the TIANGEN FastQuant RT Kit (China), incorporating a gDNA wipe buffer, and stored at − 20 °C for long-term storage.

### Candidate reference genes and primers design

Twelve common reference genes were used for this study:*18S, ACT, CYP, UBQ, UBC, TUA, GAPDH, EF-1α, MDH, PLA, TBP, and Eif-5a*. These reference sequences of these reference genes were obtained from NCBI, and specific primer pairs were designed using Primer Premier 5.0 and Oligo 7 software. All of them comes from 2 × Taq Master Mix (TsingKe, China). The reaction volume for PCR amplification was 50μL, which contained 25μL of 2 × Taq Master Mix, 19μL of ddH_2_O, 2μL of diluted template cDNA (1:5), and 2μL of each primers (10 mM). The steps involved in PCR were as follows: 5 min at 95 °C for denaturation; 35 cycles of 30 s at 95 °C (denaturation), 30 s at 60 °C (annealing), and 30 s at 72 °C (extension); and a final step of 10 min at 72 °C for extension. Every primers that was initially amplified was verified by a single PCR result that was the anticipated size according to our design. PCR products were gel-purified using the DNA Gel Extraction Kit (Axygen, USA), ligated into the pGEM-T vector (Promega, USA) using T4 DNA ligase(New England Biolabs, USA), transformed into E.coli (DH5α, TransGen Biotech, China), sequenced by Sanger sequencing (Augct, China), and compared with the reference sequence of NCBI. The consistent sequences were chosen for further study.

### Real-time PCR analysis

Real-time reverse transcription polymerase chain reaction (RT-PCR) was conducted using the StepOne Software v 2.1 an Applied Biosystems (USA) system. Each reaction consisted of 20ul, with 10ul of 2 × SuperReal PreMix Plus containing SYBR Green 1 (TIANGEN Talent qPCR PreMix, China), 4.8ul RNase-free water, 2ul of a 50 × ROX Reference Dye, 2ul of a 1:5 diluted cDNA sample, and 0.6ul of each primer (10 nM concentration). The PCR protocol involved incubating at 95 °C for 15 min, followed by 40 cycles of denaturation at 95 °C for 15 s and annealing/extension at 60 °C for 1 min, all in a 48-well plate. To ensure specificity, melting curve analysis was performed on each sample’s product. Standard curves were generated by plotting the amplification efficiency (E) and correlation coefficient (R^2^) against the serial dilutions of cDNA (5^0^, 5^–1^, 5^–2^, 5^–3^, and 5^–4^). Each RT-PCR reaction was triplicated for technical replicates, and all samples were diluted fivefold prior to the assay. Compliance with the Minimum information for publication of Quantitative Real-Time PCR Experiments (MIQE) guidelines was adhered to throughout the study^19^.

### geNorm and NormFinder algorithms software

To assess the stability of the reference genes, geNorm and NormFinder statistical methodologies were employed^20^. The qRT-PCR-derived Ct values for each sample were converted into suitable input data using the equation E^−ΔCt^, where ΔCt is the difference between the individual gene's Ct value and the minimum Ct value across all samples, as calculated by the Microsoft Excel software 2013.These data were then subjected to the algorithms for analysis. Furthermore, the relative expression levels of *UBC* and *EXPA1* genes were calculated employing the 2^−ΔCt^ formula.

### Manuscript method

The use of plant material was in accordance with relevant institutional, national, and international guidelines and legislation.

## Results

### Primers of candidate reference genes

Table 1 presents a comprehensive overview of 12 candidate reference genes (*18S*, *ACT, CYP*, *UBQ*, *UBC*, *TUA*, *GAPDH*, *EF-1α*, *MDH*, *PLA*, *TBP*, *Eif-5a*), listing their full gene names, accession numbers, primer sequences, amplicon lengths, R^2^ values of the standard curves, and primer efficiencies. Utilizing the full-length sequences retrieved from NCBI, specific primer pairs were designed and validated for their amplification specificity and efficiency. The primer pairs resulted in a single, expected PCR product ranging from 80 to 300 base pairs, as confirmed through melt curve analysis and sequencing. Following PCR amplification, all products were subjected to sequencing, which confirmed their identity as target fragments through NCBI BLAST searches. The qPCR amplifications consistently yielded Single-peak melting curves, indicative of high specificity. The primer pairs exhibited efficient amplification, with efficiency values (E) ranging from 88.998 to 100.353%, falling within the optimial range of 90–110%. The correlation coefficients (R^2^) of the standard curves varied between 0.995 and 0.999, aligning with the recommended optimal range of 0.997–0.999^21^.

**Table 1:** Candidate reference genes, primer sequences and amplicon characteristics of the lotus.

Gene symbol	Gene name	Gene accession	Sequence of forward & reverse primers	Amplicon length (bp)	R^2^	Primer efficiency
*18S*	18S ribosomal RNA	XM-010264930.1	AGTATCTCGGCAGTTCAGTG	191	0.997	92.827
			GCAAATCCAGCACGCATA			
*ACT*	β-actin	XM-010243420.1	TCGGTTGGACCTTGCT	207	0.996	100.353
			CCATCAGGCAGCTCGTA			
*CYP*	Cyclophilin	XM-010245789.1	GTACCCAGAAGAATGCCCTA	102	0.998	96.222
			ATGAAGCCCTTGATGACTCG			
*UBQ*	Ubiquitin	XM-010264087.1	TCGCACCTTGGCTGACT	281	0.996	95.465
			GGACAAGATGAAGGGTGG			
*UBC*	Ubiquitin-conjugating enzyme	XM-010246251.1	AACATCAATAGCAATGGGAGC	116	0.997	97.752
			GGATTTGGGTCCGTTAG			
*TUA*	Tubulin alpha	XM-010268491.1	GTCTTCTCCCGAATTGACCAC	86	0.998	95.173
			TCTTCCATGCCCTCGCCAA			
*GAPDHHHHHHHH*	Glyceraldehyde-3-phos -hate dehydrogenase	XM-010250569.1	CGAACAGATCAAAGCCGCTA	199	0.997	94.249
			GCTGTAACCCCATTCGTTG			
*EF-1α*	Eukaryotic elongation factor	XM-010246657.1	CAAGTCTGTTGAGATGCACCA	119	0.999	88.998
			AGTTCGAGGCAACATAACCAC			
*MDH*	Malate dehydrogenase	XM-010264019.1	CCATAACAGGGCACTAGGTCA	164	0.999	94.338
			CAACAAGCTCACGTACAGG			
*PLA*	Phospholipase A2	XM-010268730.1	TTCTCCTGTGTCCTCCTCGTC	120	0.997	91.09
			TACTCCACAGAACTTCCCGTA			
*TBP*	TATA-box-binding protein	XM-010278104.1	TTTCCAGCAAAGTTTAAGGAC	101	0.997	98.549
			GCACCATGAGAATATGCAAG			
*Eif-5a*	Eukaryotic initiation factor 5a	XM-010267296.1	GCCTTCGTCCCACAACT	94	0.998	90.252
			AGTCAGAAGGCTCACAA			

### Ct values analysis of candidate reference genes

**Figure 1:**
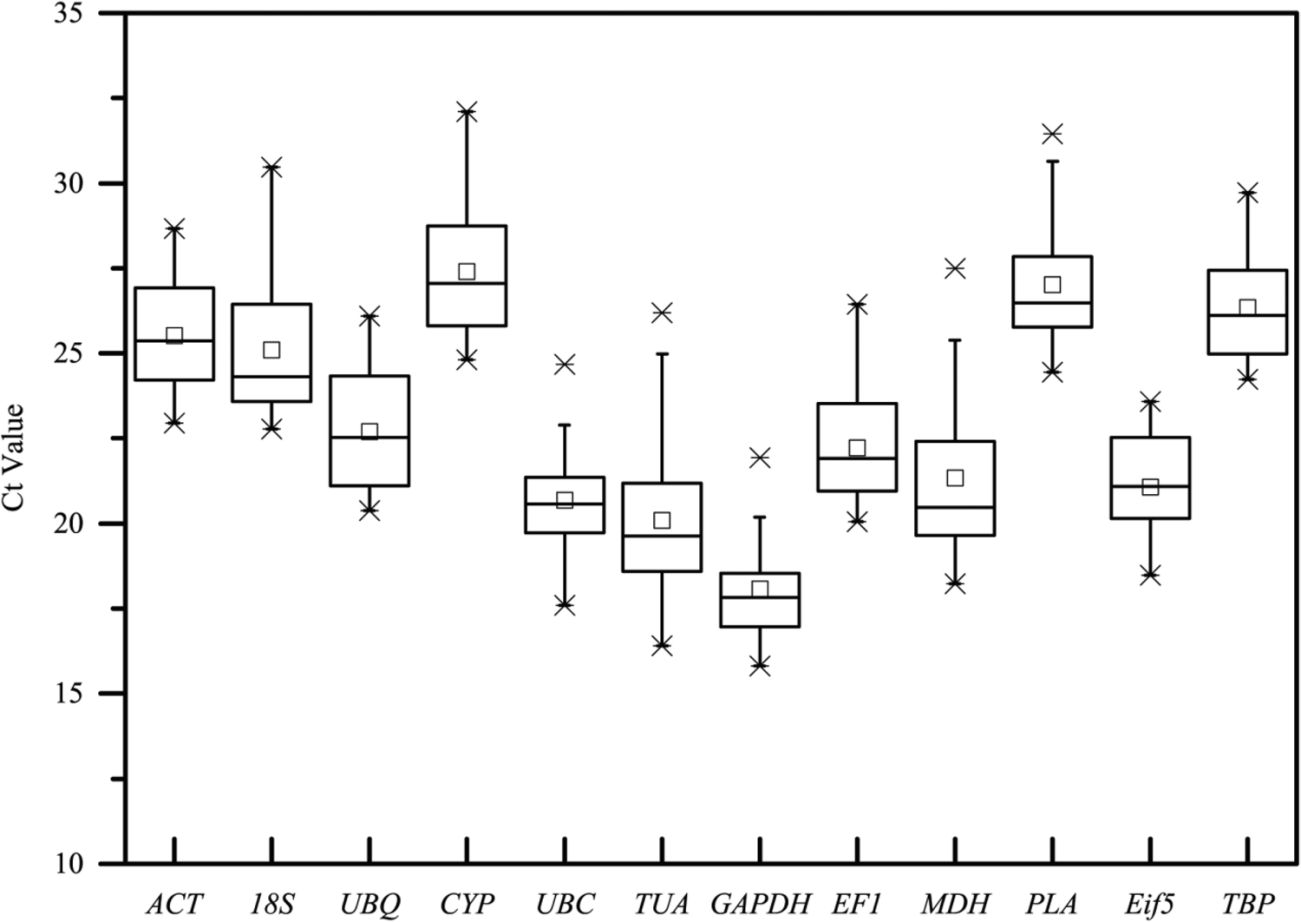
Ct mean of 12 candidate reference genes in all samples of the lotus. The Ct values were described by a Box-plot, correspond to the standard deviation. Box-plot graph of Ct values show the median values as line across the box. Lower and upper boxes indicating the first and the third quartile. Whiskers represent the maximum and minimum values. A little blot indicates a deflected data.

The initial Ct value assessment, depicted in Fig. 1 through a Box-plot, aimed to provide an overview of the reference gene abundance across all samples^22^. All automatic threshold settings were set to 1, the average value. The CT values for the 12 candidate reference genes showed a substantial variation, ranging from the lowest average of 15.812 for *GAPD*, to the highest of 32.102 for CYP in the tested lotus sample pools. Individual genes exhibited distinct expression patterns among the examined pools. Figure 1 illustrates that *UBC* exhibited the least gene expression variability, followed by *18S*, *UBQ,* and *TUA* with higher variability. Their extensive expression ranges confirmed that no single candidate reference gene maintained consistent expression under the tested conditions in lotus. The Ct values were informative, with an optimal range of 15–35 cycles for qPCR. The candidate genes' Ct values ranged from 15.8 to 32.1, all falling within the acceptable range. GAPDH, with the lowest Ct, corresponded to the highest gene expression levels, while CYP and PLA had higher Ct values, indicating lower expression. The variation in Ct values among reference genes influences qPCR accuracy. Therefore, selecting an appropriate reference gene for normalization under specific conditions in lotus is crucial.

### geNorm analysis

**Figure 2:**
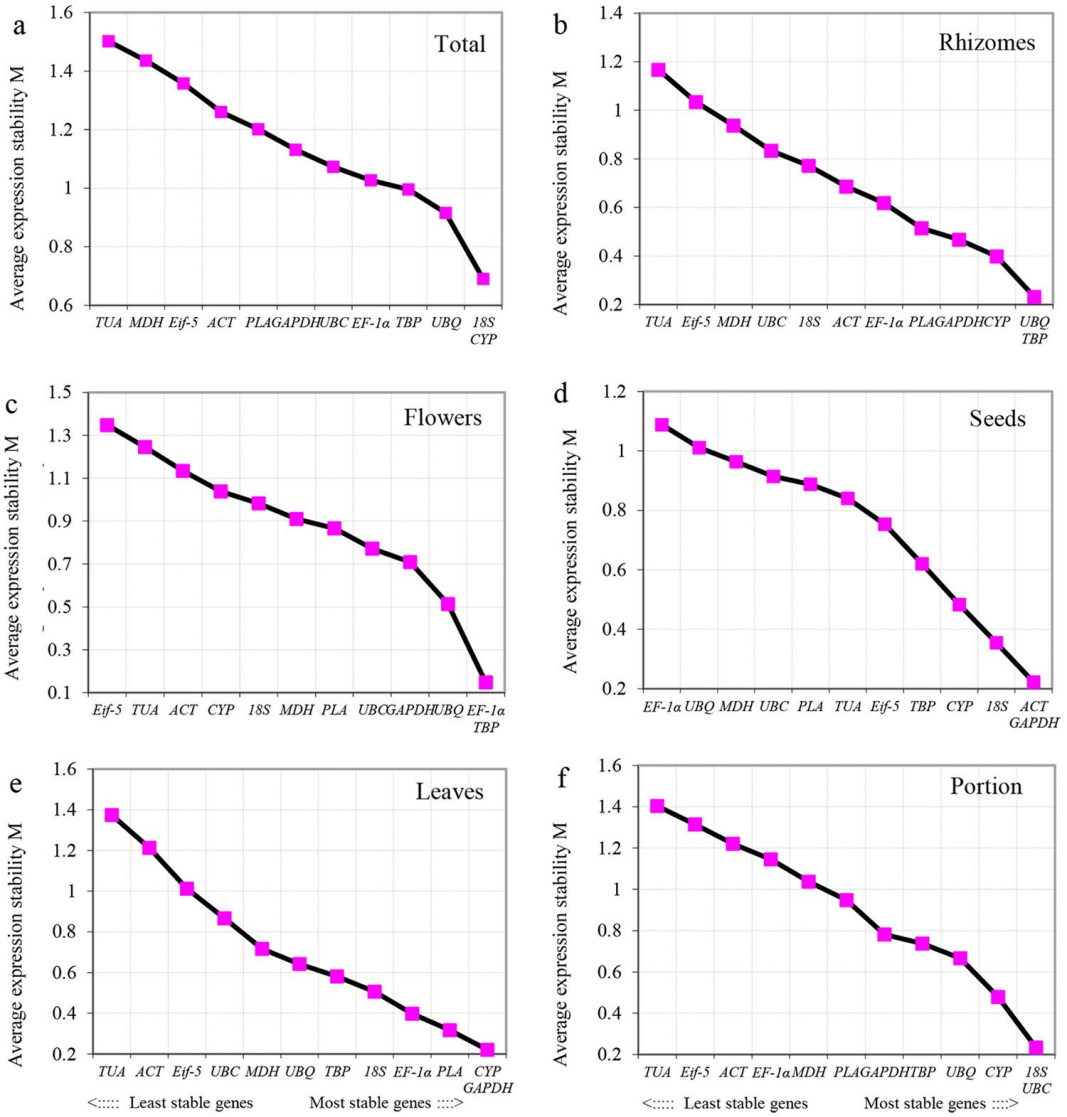
Average expression (M) values of remaining control genes of 12 candidate reference genes as calculated by geNorm. GeNorm was used to calculate the gene expression stability measure M for a reference gene. Six sets were displayed in a broken line graph, include all 18 samples pools (**a**), expanding rhizomes (**b**), different tissues of flower (**c**), different developmental stage of seeds (**d**), different developmental stage of leaves (**e**), typical tissues (**f**). The last stable genes and most stable genes are displayed from left to right, the more stable reference gene with the lower value of M.

The geNorm analysis, conducted across six series, identified the top two most stable reference genes by ranking them from least to most stable (depicted in Fig. 2). When the entire dataset of 18 samples was considered, the average expression stability (M) of *18S* and *CYP* was the lowest, followed by *UBQ*, *TBP*, and *EF-1α*, with *TUA* displaying the highest instability (Fig. 2a). This suggests that *18S* and *CYP* exhibit the most consistent expression, while *TUA* exhibits the greatest variability. The findings were consistently replicated across different tissues within the same developmental stage series. During rhizome expansion, UBQ and TBP exhibited the lowest expression stability, with TUA maintaining the least stable level (Fig. 2b). In contrast, *TBP* and *EF-1α* demonstrated consistent expression across various flower tissues, as indicated by their lowest M values (Fig. 2c). When examining seed developmental stages, *ACT* and *GAPDH* were identified as the most stable genes, while *TUA* displayed the highest variability (Fig. 2d). During leaf development, *CYP* and *GAPDH* exhibited the lowest M values*,* while *TUA* maintained the highest level of gene expression variability (Fig. 2e). When analyzing the typical tissues of lotus, geNorm suggested that *18S* and *UBC* could be appropriate reference genes (Fig. 2f). Notably, the most stable genes across the five series did not consistently overlap, although some genes, despite not being the most stable in each series, displayed lower M values in other contexts. This highlights the potential for cross-series stability in reference genes.

**Figure 3:**
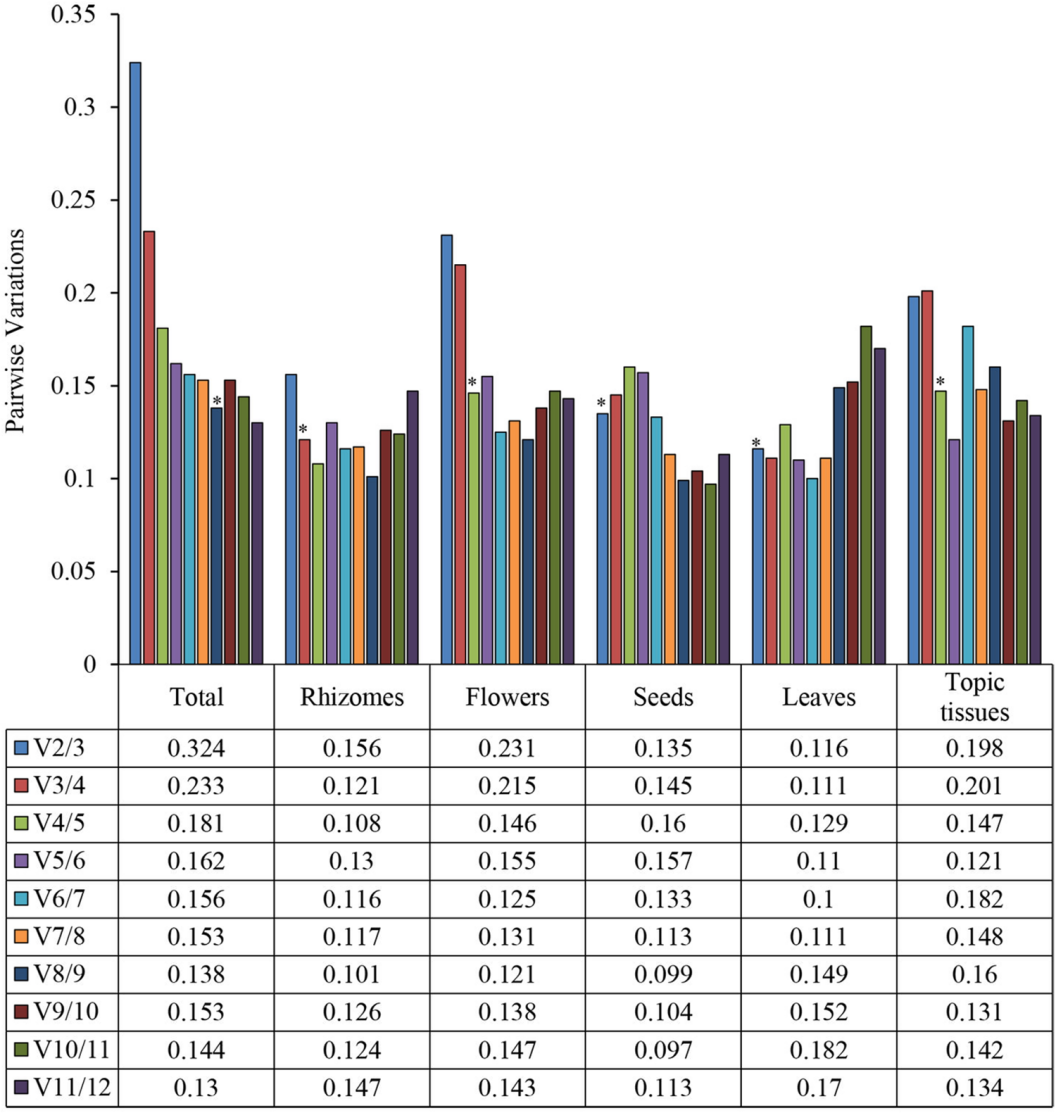
The pairwise variations of 12 reference genes calculated by geNorm. The V of six series (total, rhizomes, flowers, leaves, seeds, topic tissues) were calculated. The 0.15 is a propositional cut-off value about pairwise variation value, an extra reference gene is not required for normalization when the number is below 0.15. Pairwise variation was analyzed to determine the optimal umber of reference genes, and used * to mark propositional cut-off value.

Pairwise fluctuations (Vn/Vn + 1) between consecutive normalization factors (NFn and NFn + 1) serve as a metric to establish the optimal number of reference genes in gene expression studies. The geNorm algorithm, renowned for its precision, relies on the V value, as depicted in Fig. 3, to assess the stability of gene expression across varying conditions. The objective is to identify a set of reference genes with consistent expression profiles, ensuring reliable normalization. This study reveals that including a third reference gene in normalization had no substantial impact on pairwise variation in the rhizomes, seeds, and leaves, as indicated by the results. However, in specific tissue samples like the top and flowers, the addition of the third gene was indispensable, as the V2/3 ratio surpassed the recommended threshold of 0.15. The overall analysis necessitated the inclusion of the eighth reference gene, as V2/3, V3/4 and V4/5 exhibited values significantly greater than 1.5. Consequently, it underscores the importance of selecting multiple adaptive stable reference genes for accurate qPCR-based gene expression analysis in lotus plants.

### NormFinder analysis

The NormFinder algorithm was employed to analyze data from six distinct experimental series, with the findings presented in Table 2. Upon ranking candidate genes based on their stability value, *TBP* emerged as the top choice for overall samples. Notably, *TBP* exhibited exceptional suitability as a reference gene in the context of expanding rhizomes and developing seeds. *CYP* demonstrated superiority in various flower tissues and six standard samples, and was also highly regarded for normalization in total samples, leaves, and seeds. *GDPAH* excelled in leaves, while *ACT* outperformed others in seed samples. *TUA* displayed the highest variability in rhizomes, flowers, and typical tissues, and its variability was more pronounced in other contexts. *Eif-5a* had the highest overall variability score, indicating its potential as the most variable reference gene. *EF-1α* showed increased variability specifically in developing seeds, and *ACT ‘s* variability was observed in leaf samples.

**Table 2:** The stability value and rank of these 13 candidate reference genes were calculated from NormFinder.

Genename	Total		Rhizomes		Flowers		Seeds		Leaves		Typical tissues	
	Stability	Rank	Stability	Rank	Stability	Rank	Stability	Rank	Stability	Rank	Stability	Rank
*ACT*	0.79874	8	0.877562782	10	1.260666624	11	0.31855	1	0.790611438	12	0.821878898	8
*18S*	0.54661	2	0.704953399	8	0.56203448	7	0.36901	3	0.643991666	9	0.466743076	5
*UBQ*	0.68457	7	0.493600396	4	0.361802847	5	0.70191	10	0.599921682	8	0.417265175	3
*CYP*	0.59914	5	0.735186277	9	0.106247618	1	0.50977	6	0.360898795	3	0.221323754	1
*UBC*	0.80232	9	0.553235012	5	1.024022083	9	0.63089	9	0.589236123	7	0.379568301	2
*TUA*	1.05909	11	1.23889721	12	1.394876868	12	0.45297	5	0.705098031	10	1.091296794	12
*GAPDH*	0.54856	3	0.599310756	7	0.234372905	3	0.35111	2	0.162749072	1	0.462964466	4
*EF-1α*	0.66155	6	0.272856274	2	0.139117293	2	0.92843	12	0.346822834	2	0.973379912	10
*MDH*	1.03902	10	0.558335607	6	0.718220151	8	0.76350	11	0.717076829	11	0.880051152	9
*PLA*	0.59717	4	0.387913916	3	0.321436881	4	0.55585	8	0.411019754	6	0.758511626	7
*Eif-5*	1.06102	12	1.089021038	11	1.025516268	10	0.53138	7	0.392914835	5	1.060679762	11
*TBP*	0.35381	1	0.171712067	1	0.417067735	6	0.42525	4	0.389382501	4	0.544064367	6

### Reference gene validation

**Figure 4:**
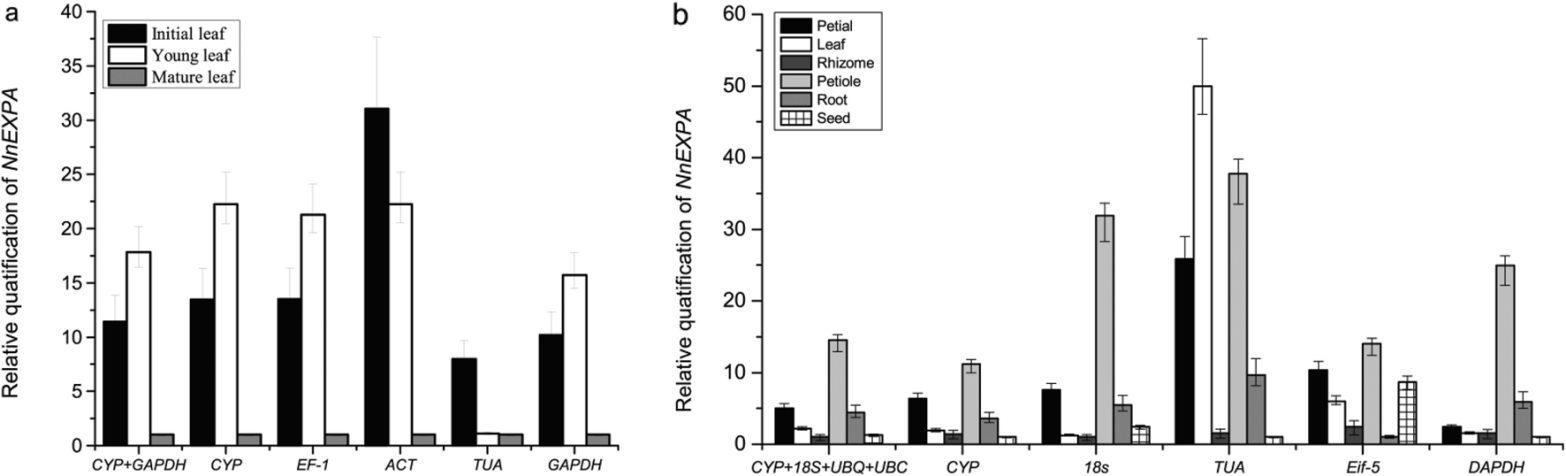
Relative quantification of *NnEXPA1* expression. *CYP*, *GAPDH*, *EF-1, ACT, TUA* and the geometric average of *CYP* + G*APDH* were used as internal controls for developing leaves (**a**); *CYP, 18S*, *TUA, Eif-5* and the geometric average of *CYP* + *18S* + *UBQ* + *UBC* were used as internal controls for tissue of lotus (**b**).

To evaluate the reliability of reference genes chosen by geNorm and NormFinder, we employed the *NnEXPA1* gene (accession No. KP322571) as an internal control, based on its relative expression levels determined by qRT-PCR. The internal control strategy involved calculating the geometric mean of the optimal gene combination from geNorm, the two most stable genes, and the least stable ones. For the developing leaves dataset, normalization was carried out using *CYP*, *ACT,* and *TUA*, with the geometric mean of *CYP* and *GAPDH* being utilized. During the analysis of tissue samples, normalization was performed using the geometric mean of *CYP*, *TUA,* and *Eif-5*, as well as *UBQ*, *TBP*, *CYP,* and *UBC*. Notably, variations in normalization based on different reference genes are illustrated in Fig. 4. EXPAs, known for their role in cell wall modification during tissue growth, exhibit high expression levels during periods of active development and tissue expansion. During leaf development, *NnEXPA1’s* relative expression displayed a rising trend, reaching approximately 1.5 times higher in young leaves compared to the initial stage, surpassing mature leaf levels, as shown in Fig. 4a. This expression pattern was determined using two internal controls and the most reliable gene, although a significant disparity was detected in the expression of the two least stable genes. In contrast, *NnEXPA1* expression was higher in the petiole and petial compared to the rhizome, with the optimal combination of reference gene capturing this variation (Fig. 4b). No significant difference in *NnEXPA1* expression was observed between *GAPDH* and the alternative candidate gene.

## Discussion

The qRT-PCR technique was considered as the gold standard for its high accuracy, real-time monitoring of reaction progression, rapid analysis, and precise quantification^23,24^. To ensure the reliability of RT-PCR data, researchers focused on selecting reference genes that are constitutively expressed at a stable and consistent level, serving as pivotal calibrators for target gene expression studies^25^. The expression patterns of the verified candidate reference genes can compensate for potential experimental errors during normalization. In this study, 12 genes (*18S, ACT, CYP, UBQ, UBC, TUA, GAPDH, EF-1α, MDH, PLA, TBP, and Eif-5a*) were cloned from lotus for use in expression normalization across 18 diverse samples. To our knowledge, no comparable report exists in the literature for lotus regarding this specific analysis.

During qRT-PCR analysis, the use of stable reference genes is crucial to minimize uncertainties across varying experimental conditions and among individuals. Consequently, extensive evaluations and validations of candidate reference genes for expression normalization have been conducted in various species. It is recognized that these genes may exhibit species-specific regulation, with differential expression patterns observed. As an example, Jain’s research highlighted the high stability of the *UBQ* and *EF-1α* genes in *Oryza sativa,* emphasizing the need for species-specific gene selection^26^. The *Coffea arabica GAPDH* gene exhibits high stability, contrasting its low stability in peach, as previously reported^27,28^. Our study employed a combined approach of software analysis and experimentation to identify the optimal reference genes. The results consistently ranked *CYP* as the top choice across various conditions, followed by *GAPDH*, *TBP,* and *18S*. *ACT* often regarded as a Housekeeping gene in lotus gene expression studies. surprisingly displayed instability in both our tested samples and across experimental setups, falling short of expectations.

We employed geNorm and NormFinder software to analyze the data, revealing discrepancies in stability rankings and coherence outcomes between the two algorithms. While TBP was deemed the most stable gene for the total sample pool by NormFinder, this was not the case for geNorm. In rhizomes, both geNorm and NormFinder concurred that TBP was the optimal reference gene. ACT demonstrated higher stability across seed samples according to both geNorm and NormFinder, but its stability varied in other experimental conditions. GeNorm operates on the assumption that the expression ratio of ideal reference genes remains constant across all samples, independent of experimental conditions or cell types. Stability is determined by the lowest M value, indicating the most stable gene, while the highest M signifies least stability. In certain experimental scenarios, a single reliable internal control gene may not exist, necessitating the use of one or more reference genes for precise normalization to ensure accurate result^6^. The two most stable genes were identified as the optimal choice for their average expression stability (M) values.

To verify the reliability of the previously selected reference genes, *NnEXPA1* was chosen for expression analysis. *NnEXPA1,* a member of the EXPA (α-expansin) subfamily, is associated with EXPA proteins that play a crucial role in cell wall loosening and cell expansion, contributing to various plant developmental processes such as internode elongation, root growth^29^, seed development^30^, endosperm expansion^31^, and nodule formation^32^. When employed as internal controls with different reference genes tailored to specific conditions, no significant expression discrepancies were detected among the recommended candidates. The results were validated, and we observed no significant expression difference in *NnEXPA1* when compared to *GAPDH* and other candidates. This aligns with our expectations, as it suggests that multiple candidate genes are suitable for gene expression in lotus^33^.This finding highlights that a low Ct value for a reference gene does not guarantee the detection of minute gene expression variations. Consequently, it underscores the significance of selecting appropriate reference genes for obtaining precise and reliable qPCR outcomes.

The advent of the genomic era has witnessed a surge in gene expression studies on lotus, with the proliferation of gene expression chips and the expansion of EST (Expressed Sequence Tags) databases. were reported. This progress has expanded the repertoire of reference genes in lotus beyond the conventional housekeeping genes,introducing a more robust and inclusive set of genes that exhibit higher stability and broader coverage for accurate transcriptome analysis.

## Data Availability

All data generated or analysed during this study are included in this published article.

## Abbreviations

qRT-PCR: Quantitative real-time reverse transcription polymerase chain reaction
HKG: House-keeping gene
MIQE: Minimum information for publication of quantitative real-time PCR experiments

## References

[1] Yanjing R, Rui H, Mengliang Z (2021). Internal reference genes screening of turnip by real-time fluorescence quantiative PCR. Qinghai Agric. For. Sci. Technol..

[2] Mafra V (2012). Reference genes for accurate transcript normalization in citrus genotypes under different experimental conditions. PLoS ONE.

[3] Wang M, Bhullar NK (2021). Selection of suitable reference genes for qRT-PCR gene expression studies in rice. Methods Mol. Biol..

[4] Radonic A, Thulke S, Mackay IM, Landt O, Siegert W, Nitsche A (2004). Guideline to reference gene selection for quantitative real-time PCR. Biochem. Biophys. Res. Commun..

[5] Vandesompele J, De Preter K, Pattyn F, Poppe B, Van Roy N, De Paepe A, Speleman F (2002). Accurate normalization of real-time quantitative RT-PCR data by geometric averaging of multiple internal control genes. Genome Biol..

[6] Rohit B (2022). Selection and validation of the most suitable reference genes for quantitative real-time PCR normalization in salvia rosmarinus under in vitro conditions. Plants (Basel)..

[7] Horgusluoglu E, Neff R, Song WM (2022). Integrative metabolomics-genomics approach reveals key metabolic pathways and regulators of Alzheimer's disease. Alzheimers Dement..

[8] Li F, Cheng Y, Ma L (2022). Identification of reference genes provides functional insights into meiotic recombination suppressors in Gerbera hybrida. Horticult. Plant J..

[9] Mingming Y, Jing Z, Jieying X (2020). Screening of internal reference genes after rhesus monkey kidney cells are infected with human parainfluenza virus 2. Chongqing Med..

[10] Diao Y (2016). Cloning and characterization of the UBC gene from lotus (Nelumbo nucifera). Genet. Mol. Res..

[11] Yinjie W (2021). Selection and validation of appropriate reference genes for RT-qPCR analysis of flowering stages and different genotypes of Iris germanica L. Sci. Rep..

[12] Hui Z (2022). Systematic identification and validation of suitable reference genes for the normalization of gene expression in prunella vulgaris under different organs and spike development stages. Genes (Basel).

[13] Peirong Xu, Minzheng Li (2021). A review of lotus germplasm resources. Agric. Technol..

[14] Bangar SP (2022). A comprehensive review on lotus seeds (Nelumbo nucifera Gaertn): Nutritional composition, health-related bioactive properties, and industrial applications. J. Funct. Foods..

[15] Hui Li (2021). Nelumbo genome database, an integrative resource for gene expression and variants of Nelumbo nucifera. Sci. Data..

[16] Tao S (2022). Advances and Prospects in Genomic and Functional Studies of the Aquatic Crop, Sacred Lotus.

[17] Zhiyan G (2022). Genome-wide association study of traits in sacred lotus uncovers MITE-associated variants underlying stamen petaloid and petal number variations. Front Plant Sci..

[18] Ping Z (2022). Comparative analyses of American and Asian lotus genomes reveal insights into petal color, carpel thermogenesis and domestication. Plant J..

[19] Wei Y, Liu Y, Li L, Xiang S, Zhang H, Shang Y (2022). Identification of s9ap used as an endogenous reference gene in qualitative and real-time quantitative PCR detection of Pleurotus eryngii. Mol. Biol. Rep..

[20] Yang Z, Zhang R, Zhou Z (2021). Identification and validation of reference genes for gene expression analysis in *Schima*
*superba*. Genes (Basel)..

[21] XiaoZhe Yi (2022). Screening of reference genes for quantitative real-time PCR in Artemisia argyi. Zhongguo Zhong Yao Za Zhi..

[22] Ramirez-Hinojosa JP (2021). Association between cycle threshold (Ct) values and clinical and laboratory data in inpatients with COVID-19 and asymptomatic health workers. J. Med. Virol..

[23] Li Y (2023). Genome-wide identification and expression analysis of NAC family genes in Ginkgo biloba L. Plant Biol. (Stuttg)..

[24] Ying Yu (2021). Identification and characterization of differentially expressed microRNAs and target gene related to flax stem development. J. Natl. Fibers.

[25] Dylan P, Christian P, WooSuk C (2022). Identification and validation of reference genes for expression analysis in nitrogen-fixing bacteria under environmental stress. Life (Basel)..

[26] Williams ML, Ghanem M (2022). Evaluation of candidate reference genes stability for gene expression analysis by reverse transcription qPCR in Clostridium perfringens. Sci. Rep..

[27] Yang Y (2022). Genome-wide screening and stability verification of the robust internal control genes for RT-qPCR in filamentous fungi. J. Fungi.

[28] Ping Z (2018). GAPDH rs1136666 SNP indicates a high risk of Parkinson’s disease. Neurosci. Lett..

[29] Yao Z, Wang Z, Fang B (2020). Involvement of nitrogen in storage root growth and related gene expression in sweet potato (Ipomoea batatas). Plant Biol. (Stuttg)..

[30] Bhagwat N (2022). Longer duration of active oil biosynthesis during seed development is crucial for high oil yield—lessons from genome-wide in silico mining and RNA-Seq validation in sesame. Plants (Basel).

[31] Chunmei He (2020). Overexpression of an antisense RNA of maize receptor-like kinase gene ZmRLK7 enlarges the organ and seed size of transgenic arabidopsis plants. Front Plant Sci..

[32] Thomas J, Hiltenbrand R, Bowman MJ (2020). Time-course RNA-seq analysis provides an improved understanding of gene regulation during the formation of nodule-like structures in rice. Plant Mol. Biol..

[33] Rundong Y (2022). Selection and identification of a reference gene for normalizing real-time PCR in mangos under various stimuli in different tissues. Horticulturae.

